# Wounds of Joints

**Published:** 1868-12

**Authors:** 


Wounds of Joints.—A short but interesting discussion took place at a late meeting of the Paris Society of Surgery, on the subject of the treatment of wounds penetrating joints. The question raised was the following:

What is the best method of treating those grave inflammatory accidents which so frequently result from penetrating wounds of joints?

Surgeons take credit for being able to give a more positive diagnosis, and for possessing more certain principles of treatment for their external maladies, than physicians can boast of for their internal ones.

Some surgeons, with M. Verneuil, are in favor of excision of the articular surfaces.

Others, including MM. Chassaignac and Alphonse Guerin, prefer the use of drainage.

Others, with M. Blot, think that very often the unaided efforts of nature are sufficient to obtain a cure, and recommend expectant treatment.

Others, and these are the majority, hold that it is difficult to lay down a general rule which shall be applicable to all cases, and that it is necessary to take into account, not only the amount of injury, but the joint injured, and the state of the patient's constitution.

Notwithstanding all these varying opinions, one great principle characterizes all the tendencies of modern surgery, which may be very well expressed in the words, *Conservative Surgery.*

We frequently find that the immediate closure of the wound, absolute rest of the limb, and continued irrigations, exorcise the phenomena of inflammation, and rapidly accomplish a cure. But, again, these methods may fail. Are there no others? We are convinced, for our part, that the process of pneumatic occlusion is suited to render in these cases the very greatest services. This process, indeed, fulfills the triple indication arrived at in the treatment of joint wounds.

1. It keeps the wound closed and opposes the entrance of air. 2. By the compression it exercises, it contributes to prevent and to combat the inflammatory congestion or enlargement of the tissues. 3. By its suction power it completes (if suppuration has not been prevented) the indication of drainage, in opposing in a most active manner the stagnation of liquids.—*Gazette Medicale.*

